# Di-μ-chlorido-bis­[chlorido(1,4,6-trimethyl-6-nitro-1,4-diazepine)copper(II)]

**DOI:** 10.1107/S1600536808043390

**Published:** 2009-01-08

**Authors:** Adailton J. Bortoluzzi, Ademir Neves, Rosely A. Peralta, Tiago P. Camargo, Vitor C. Weiss

**Affiliations:** a Depto. de Química–UFSC, 88040-900 Florianópolis, SC, Brazil

## Abstract

The title neutral copper complex, [Cu_2_Cl_4_(C_8_H_17_N_3_O_2_)_2_], shows a binuclear center with a Cu—(μ-Cl)_2_—Cu core, in which each copper ion is coordinated by the *N*,*N*,*O* donor atoms of the tridentate ligand 1,4,6-trimethyl-6-nitro-1,4-diazepine (meaaz-NO_2_) and three chloride exogenous ligands. Each metal ion is facially coordinated by meaaz-NO_2_ through *N*,*N*,*O* donor atoms, whereas two bridging and one terminal chloride ions occupy the other face of the highly Jahn–Teller-distorted octa­hedron. Two N atoms from tertiary amine groups of the meaaz-NO_2_ ligand and two exogenous Cl atoms with short Cu—N and Cu—Cl distances define the equatorial plane. The coordination around each Cu^II^ ion is completed by another Cl atom and an O atom from the NO_2_ group, in the axial positions. The binuclear complex exhibits a centrosymmetric structure with point symmetry 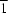.

## Related literature

For related literature, see: Belousoff *et al.* (2006 ▶); Deal & Burstyn (1996 ▶); Fry *et al.* (2005 ▶); Hegg & Burstyn (1998 ▶); Peralta *et al.* (2005 ▶); Rodriguez, *et al.* (1999 ▶); Romba *et al.* (2006 ▶). For the synthesis of the meaaz-NO_2_ ligand see Ge *et al.* (2006 ▶). For related structures, see: Astner *et al.* (2008 ▶); Schwindinger *et al.* (1980 ▶); Steed *et al.* (2007 ▶).
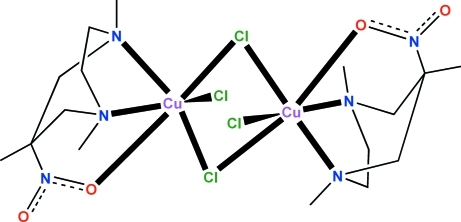


## Experimental

### 

#### Crystal data

[Cu_2_Cl_4_(C_8_H_17_N_3_O_2_)_2_]
*M* *_r_* = 643.37
Monoclinic, 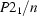

*a* = 10.5478 (2) Å
*b* = 10.9251 (2) Å
*c* = 11.4430 (2) Å
β = 102.297 (1)°
*V* = 1288.39 (4) Å^3^
*Z* = 2
Mo *K*α radiation
μ = 2.10 mm^−1^
*T* = 296 (2) K
0.31 × 0.14 × 0.09 mm

#### Data collection

Bruker APEXII CCD area-detector diffractometer
Absorption correction: multi-scan (*SADABS*; Bruker, 2006 ▶) *T*_min_ = 0.562, *T*_max_ = 0.833
25284 measured reflections
2528 independent reflections
2080 reflections with *I* > 2σ(*I*)
*R*_int_ = 0.036

#### Refinement

*R*[*F* ^2^ > 2σ(*F* ^2^)] = 0.030
*wR*(*F* ^2^) = 0.083
*S* = 1.07
2528 reflections
148 parameters
H-atom parameters constrained
Δρ_max_ = 0.76 e Å^−3^
Δρ_min_ = −0.37 e Å^−3^

### 

Data collection: *APEX2*, *BIS* and *COSMO* (Bruker, 2006 ▶); cell refinement: *SAINT* (Bruker, 2006 ▶); data reduction: *SAINT*; program(s) used to solve structure: *SHELXTL* (Sheldrick, 2008 ▶); program(s) used to refine structure: *SHELXL97* (Sheldrick, 2008 ▶); molecular graphics: *PLATON* (Spek, 2003 ▶); software used to prepare material for publication: *SHELXL97*.

## Supplementary Material

Crystal structure: contains datablocks global, I. DOI: 10.1107/S1600536808043390/pk2138sup1.cif

Structure factors: contains datablocks I. DOI: 10.1107/S1600536808043390/pk2138Isup2.hkl

Additional supplementary materials:  crystallographic information; 3D view; checkCIF report

## Figures and Tables

**Fig. 1.**
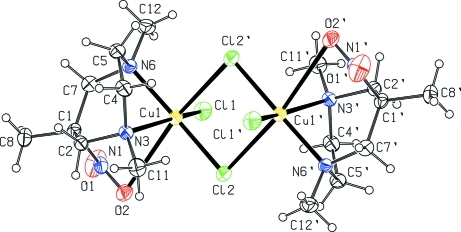
A view of the molecular structure of copper complex showing the atom-labelling scheme. Ellipsoids are drawn at the 30% probability level. H atoms are omitted for clarity.

**Fig. 2.**
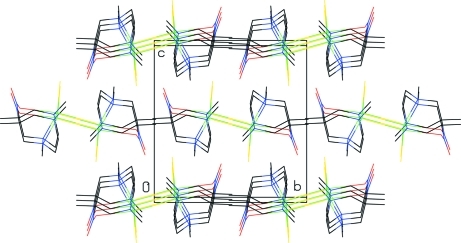
View down the a axis of the packing of copper complex.

## supplementary crystallographic information

### Comment

Tridentate ligands that are able to force facial geometry, such as 1,4,7-tacn (1,4,7-triazacyclononane), daza (1,4-diazepan-6-amine) (Romba, *et al.*, 2006), tach (*cis*,*cis*-1,3,5-triaminocyclohexane) (Hegg & Burstyn, 1998), play an important role in the stabilization of a great number of structural motifs in coordination compounds and in biological systems (Peralta *et al.*, 2005). Copper complexes with this kind of ligand have been reported over the past few years with a view to the study of the hydrolysis of phosphate esters, proteins and DNA (Deal & Burstyn, 1996; Fry *et al.*, 2005). Indeed such copper(II) complexes exhibit high catalytic reactivity in the hydrolysis of DNA model diesters as bis(4-nitrophenyl)phosphate with rate constants of ≈ 10 ^-4^ s^-1^ (Belousoff *et al.*, 2006). In this context we report herein the synthesis and X-ray analysis of a new dinuclear copper complex with the tridentate ligand meaaz-NO_2_.

This neutral copper complex exhibits a centrosymmetric structure (Fig. 1) with a highly distorted octahedral environment around the copper center. Each metal ion is facially coordinated by meaaz-NO_2_ through N_2_O donors atoms, whereas two bridged and one terminal coordinated chlorines occupy the other face of the distorted octahedron. Two amine nitrogen atoms (N3 and N6) of the ligand and two exogenous chlorines (Cl1, Cl2) lie in the equatorial plane. The coordination sphere of Cu1 is completed by another chlorine (Cl2') and an oxygen atom (O2) from the NO_2_ group, in the axial positions. In the equatorial plane, the Cu—N and Cu—Cl bond lengths are Cu1—N6 2.064 (2) Å, Cu1—N3 2.122 (2) Å, Cu1—Cl2 2.2686 (7) Å and Cu1—Cl1 2.2694 (7) Å), respectively. The longer bond lengths Cu1—Cl2' (2.7611 (8) Å) and Cu1—O2 (2.845 (2) Å) are associated with the two apical positions, as expected for a (4 + 2) distorted geometry, as is common for Cu^II^.  The Cu—N and the Cu—Cl bond lengths in the equatorial plane are comparable to those found for other copper complexes [Cu(tacn)Cl_2_] (Cu—N2 2.063 (4) Å, Cu—N3 2.038 (4) Å, CU—Cl1 2.268 (1) Å and Cu—Cl2 2.312 (1) Å) (Schwindinger, *et al.*, 1980), [Cu_2_(µ-Cl)_2_(Me-bpa)_2_(ClO_4_)_2_] (Me-bpa = *N*-methyl-bis(2-pyridylmethyl)amine) (Cu1—N36 1.983 (2) Å, Cu1—N10 2.036 (2) Å, Cu1—N26 1.989 (2) Å and Cu1—Cl1 2.2587 (6) Å) (Astner *et al.*, 2008) and [Cu(me_3_tacn)Cl_2_] (Cu1—N1 2.100 (2) Å, Cu1—N2 2.111 (2) Å, Cu1—Cl2 2.2558 (9) Å and Cu1—Cl1 2.3050 (8) Å) (Steed *et al.*, 2007). The seven-membered chelate ring of the meaaz-NO_2_ ligand restricts the N—Cu—N angle to 77.35 (8)°, which is about 6° smaller than the respective angles formed by nine-membered ring in the Cu-tacn complexes.

As described in Rodriguez, *et al.* (1999), there are three kinds of configurations for copper complex containing the Cu-(µ-Cl)_2_—Cu core: Type I, in which two square pyramids share one base-to-apex edge with the two bases nearly perpendicular to one another; Type II, square pyramids sharing one base-to-apex edge but with parallel basal planes and Type III, square pyramids sharing a basal edge with coplanar basal planes. The configuration of the Cu centers reported here are Type II given that the axial positions of one copper(II) center is directed toward the top and the same axis of the adjacent center is in the anti position. Although the Cu centers in the complex are hexacoordinate, it can be considered as type II, because the sixth coordination bond is very long (Cu—O2 = 2.845 (2)Å).

The packing is mainly governed by weak C—H···O and C—H···Cl interactions with average D···A distances of 2.99 Å and 3.74 Å, respectively. In addition, the packing analysis reveals that the molecules are accomodated in layers parallel to the (001) plane and are stacked along crystallographic *a* axis (Fig. 2).

### Experimental

The ligand 6-nitro-1,4,7-trimethyl-1,4-diazepine (meaaz-NO_2_) was prepared as reported in the literature (Ge *et al.*, 2006). The ligand was obtained with good yeld and was characterized by ^1^H NMR [δ (p.p.m.) (CDCl3) 400 MHz: 1.46 (s, 3H); 2.36 (s, 6H); 2.48 (m, 2H); 2.56 (m, 2H); 2.66 and 3.36 (AB system, 4H)].

Copper complex was synthesized by adding 187 mg of the ligand meaaz-NO_2_ (1.0 mmol) to a CH_3_CN solution containing CuCl_2_.2H_2_O (171 mg, 1.0 mmol). The solution was then concentrated under magnetic stirring and was allowed to stand at room temperature for a few days, yielding a small number of dark green crystals which were suitable for the single-crystal X-ray analysis.

### Refinement

H atoms were placed at their idealized positions with distances of 0.97 and 0.96 Å and *U*_eq_ fixed at 1.2 and 1.5 times *U*_iso_ of the preceding atom for CH_2_ and CH_3_, respectively.

### Crystal data

**Table:**

[Cu_2_Cl_4_(C_8_H_17_N_3_O_2_)_2_]	*F*(000) = 660
*M**_r_* = 643.37	*D*_x_ = 1.658 Mg m^−^^3^
Monoclinic, *P*2_1_/*n*	Mo *K*α radiation, λ = 0.71073 Å
Hall symbol: -P 2yn	Cell parameters from 6584 reflections
*a* = 10.5478 (2) Å	θ = 2.6–29.9°
*b* = 10.9251 (2) Å	µ = 2.10 mm^−^^1^
*c* = 11.4430 (2) Å	*T* = 296 K
β = 102.297 (1)°	Block, dark green
*V* = 1288.39 (4) Å^3^	0.31 × 0.14 × 0.09 mm
*Z* = 2	

### Data collection

**Table:**

Bruker APEXII CCD area-detector diffractometer	2528 independent reflections
Radiation source: fine-focus sealed tube	2080 reflections with *I* > 2σ(*I*)
graphite	*R*_int_ = 0.036
φ and ω scans	θ_max_ = 26.0°, θ_min_ = 2.7°
Absorption correction: multi-scan (*SADABS*; Bruker, 2006)	*h* = −13→12
*T*_min_ = 0.562, *T*_max_ = 0.833	*k* = −13→13
25284 measured reflections	*l* = −14→14

### Refinement

**Table:**

Refinement on *F*^2^	Primary atom site location: structure-invariant direct methods
Least-squares matrix: full	Secondary atom site location: difference Fourier map
*R*[*F*^2^ > 2σ(*F*^2^)] = 0.030	Hydrogen site location: inferred from neighbouring sites
*wR*(*F*^2^) = 0.083	H-atom parameters constrained
*S* = 1.07	*w* = 1/[σ^2^(*F*_o_^2^) + (0.0432*P*)^2^ + 0.7796*P*] where *P* = (*F*_o_^2^ + 2*F*_c_^2^)/3
2528 reflections	(Δ/σ)_max_ = 0.001
148 parameters	Δρ_max_ = 0.76 e Å^−^^3^
0 restraints	Δρ_min_ = −0.37 e Å^−^^3^

### Special details

**Table:**

Experimental. Absorption correction: SADABS (Bruker, 2006) was used to scale the data and to perform the multi-scan semi-empirical absorption correction.

### Fractional atomic coordinates and isotropic or equivalent isotropic displacement parameters (Å^2^)

**Table:**

	*x*	*y*	*z*	*U*_iso_*/*U*_eq_	
Cu1	0.41672 (3)	0.13540 (3)	1.03616 (3)	0.03146 (12)	
Cl1	0.45369 (7)	0.11762 (7)	1.23819 (6)	0.0467 (2)	
Cl2	0.62792 (6)	0.10672 (6)	1.02889 (7)	0.04311 (19)	
N1	0.4017 (2)	0.4025 (2)	1.0951 (2)	0.0379 (5)	
C1	0.2738 (2)	0.3853 (2)	0.9997 (2)	0.0328 (6)	
C2	0.3105 (2)	0.3551 (2)	0.8818 (2)	0.0323 (5)	
H2A	0.2344	0.3665	0.8182	0.039*	
H2B	0.3752	0.4138	0.8689	0.039*	
N3	0.3623 (2)	0.22930 (18)	0.87072 (18)	0.0305 (5)	
C4	0.2543 (3)	0.1488 (2)	0.8105 (2)	0.0385 (6)	
H4A	0.2887	0.0705	0.7919	0.046*	
H4B	0.2099	0.1862	0.7361	0.046*	
C5	0.1594 (3)	0.1294 (2)	0.8915 (2)	0.0384 (6)	
H5A	0.0839	0.1809	0.8647	0.046*	
H5B	0.1307	0.0448	0.8860	0.046*	
N6	0.2196 (2)	0.15908 (19)	1.01947 (18)	0.0318 (5)	
C7	0.1930 (3)	0.2888 (3)	1.0467 (2)	0.0392 (6)	
H7A	0.2071	0.2978	1.1329	0.047*	
H7B	0.1021	0.3056	1.0139	0.047*	
C8	0.2051 (3)	0.5099 (3)	0.9930 (3)	0.0437 (7)	
H8A	0.2607	0.5721	0.9723	0.065*	
H8B	0.1857	0.5286	1.0693	0.065*	
H8C	0.1260	0.5068	0.9333	0.065*	
O2	0.5045 (2)	0.38177 (18)	1.0687 (2)	0.0487 (5)	
O1	0.3919 (3)	0.4391 (3)	1.1928 (2)	0.0739 (8)	
C11	0.4596 (3)	0.2357 (3)	0.7947 (3)	0.0458 (7)	
H11A	0.4905	0.1548	0.7834	0.069*	
H11B	0.5310	0.2860	0.8330	0.069*	
H11C	0.4204	0.2703	0.7185	0.069*	
C12	0.1585 (3)	0.0810 (3)	1.0982 (3)	0.0480 (7)	
H12A	0.1948	0.1003	1.1803	0.072*	
H12B	0.1745	−0.0036	1.0837	0.072*	
H12C	0.0667	0.0957	1.0815	0.072*	

### Atomic displacement parameters (Å^2^)

**Table:**

	*U*^11^	*U*^22^	*U*^33^	*U*^12^	*U*^13^	*U*^23^
Cu1	0.02805 (18)	0.03469 (19)	0.02830 (19)	0.00043 (12)	−0.00153 (12)	0.00140 (13)
Cl1	0.0457 (4)	0.0598 (5)	0.0286 (3)	−0.0011 (3)	−0.0057 (3)	0.0030 (3)
Cl2	0.0305 (3)	0.0414 (4)	0.0548 (4)	0.0014 (3)	0.0034 (3)	−0.0005 (3)
N1	0.0431 (14)	0.0303 (12)	0.0341 (13)	0.0022 (10)	−0.0052 (10)	−0.0050 (10)
C1	0.0286 (13)	0.0343 (14)	0.0307 (13)	0.0012 (10)	−0.0043 (10)	−0.0038 (10)
C2	0.0350 (13)	0.0290 (13)	0.0291 (13)	−0.0018 (10)	−0.0014 (10)	0.0010 (10)
N3	0.0340 (11)	0.0295 (11)	0.0264 (10)	−0.0021 (8)	0.0026 (8)	−0.0009 (9)
C4	0.0474 (16)	0.0340 (15)	0.0280 (13)	−0.0064 (11)	−0.0059 (11)	−0.0032 (11)
C5	0.0362 (14)	0.0385 (15)	0.0339 (14)	−0.0074 (11)	−0.0071 (11)	−0.0005 (11)
N6	0.0291 (11)	0.0348 (12)	0.0290 (11)	−0.0026 (9)	0.0004 (9)	0.0036 (9)
C7	0.0367 (14)	0.0420 (16)	0.0389 (15)	0.0005 (11)	0.0080 (12)	−0.0009 (12)
C8	0.0431 (16)	0.0391 (15)	0.0450 (16)	0.0096 (12)	0.0008 (13)	−0.0041 (13)
O2	0.0340 (11)	0.0482 (12)	0.0561 (13)	−0.0006 (8)	−0.0078 (9)	−0.0021 (10)
O1	0.0730 (17)	0.095 (2)	0.0429 (13)	0.0211 (14)	−0.0124 (11)	−0.0325 (13)
C11	0.0544 (18)	0.0485 (17)	0.0381 (15)	0.0034 (14)	0.0176 (13)	0.0033 (13)
C12	0.0418 (16)	0.0530 (18)	0.0499 (18)	−0.0078 (13)	0.0114 (13)	0.0137 (15)

### Geometric parameters (Å, °)

**Table:**

Cu1—N6	2.064 (2)	C4—H4A	0.9700
Cu1—N3	2.122 (2)	C4—H4B	0.9700
Cu1—Cl2	2.2686 (7)	C5—N6	1.502 (3)
Cu1—Cl1	2.2694 (7)	C5—H5A	0.9700
Cu1—Cl2^i^	2.7611 (8)	C5—H5B	0.9700
Cu1—O2	2.845 (2)	N6—C12	1.484 (3)
Cl2—Cu1^i^	2.7611 (8)	N6—C7	1.491 (3)
N1—O2	1.207 (3)	C7—H7A	0.9700
N1—O1	1.212 (3)	C7—H7B	0.9700
N1—C1	1.556 (3)	C8—H8A	0.9600
C1—C2	1.517 (4)	C8—H8B	0.9600
C1—C7	1.524 (4)	C8—H8C	0.9600
C1—C8	1.536 (4)	C11—H11A	0.9600
C2—N3	1.495 (3)	C11—H11B	0.9600
C2—H2A	0.9700	C11—H11C	0.9600
C2—H2B	0.9700	C12—H12A	0.9600
N3—C11	1.482 (3)	C12—H12B	0.9600
N3—C4	1.487 (3)	C12—H12C	0.9600
C4—C5	1.517 (4)		
			
N6—Cu1—N3	77.35 (8)	N3—C4—H4B	109.7
N6—Cu1—Cl2	172.72 (6)	C5—C4—H4B	109.7
N3—Cu1—Cl2	96.62 (6)	H4A—C4—H4B	108.2
N6—Cu1—Cl1	93.22 (6)	N6—C5—C4	111.6 (2)
N3—Cu1—Cl1	154.87 (6)	N6—C5—H5A	109.3
Cl2—Cu1—Cl1	93.93 (3)	C4—C5—H5A	109.3
N6—Cu1—Cl2^i^	89.11 (6)	N6—C5—H5B	109.3
N3—Cu1—Cl2^i^	102.97 (6)	C4—C5—H5B	109.3
Cl2—Cu1—Cl2^i^	88.29 (2)	H5A—C5—H5B	108.0
Cl1—Cu1—Cl2^i^	100.08 (3)	C12—N6—C7	107.1 (2)
N6—Cu1—O2	100.77 (7)	C12—N6—C5	108.6 (2)
N3—Cu1—O2	71.22 (7)	C7—N6—C5	110.5 (2)
Cl2—Cu1—O2	80.84 (5)	C12—N6—Cu1	115.53 (16)
Cl1—Cu1—O2	88.12 (5)	C7—N6—Cu1	109.30 (15)
Cl2^i^—Cu1—O2	166.84 (5)	C5—N6—Cu1	105.75 (16)
Cu1—Cl2—Cu1^i^	91.71 (2)	N6—C7—C1	116.1 (2)
O2—N1—O1	123.4 (2)	N6—C7—H7A	108.3
O2—N1—C1	119.5 (2)	C1—C7—H7A	108.3
O1—N1—C1	117.1 (2)	N6—C7—H7B	108.3
C2—C1—C7	115.6 (2)	C1—C7—H7B	108.3
C2—C1—C8	110.8 (2)	H7A—C7—H7B	107.4
C7—C1—C8	109.7 (2)	C1—C8—H8A	109.5
C2—C1—N1	107.6 (2)	C1—C8—H8B	109.5
C7—C1—N1	107.6 (2)	H8A—C8—H8B	109.5
C8—C1—N1	104.9 (2)	C1—C8—H8C	109.5
N3—C2—C1	116.3 (2)	H8A—C8—H8C	109.5
N3—C2—H2A	108.2	H8B—C8—H8C	109.5
C1—C2—H2A	108.2	N1—O2—Cu1	85.76 (15)
N3—C2—H2B	108.2	N3—C11—H11A	109.5
C1—C2—H2B	108.2	N3—C11—H11B	109.5
H2A—C2—H2B	107.4	H11A—C11—H11B	109.5
C11—N3—C4	108.3 (2)	N3—C11—H11C	109.5
C11—N3—C2	108.6 (2)	H11A—C11—H11C	109.5
C4—N3—C2	109.0 (2)	H11B—C11—H11C	109.5
C11—N3—Cu1	117.18 (17)	N6—C12—H12A	109.5
C4—N3—Cu1	99.36 (15)	N6—C12—H12B	109.5
C2—N3—Cu1	113.78 (15)	H12A—C12—H12B	109.5
N3—C4—C5	109.8 (2)	N6—C12—H12C	109.5
N3—C4—H4A	109.7	H12A—C12—H12C	109.5
C5—C4—H4A	109.7	H12B—C12—H12C	109.5

Symmetry codes: (i) −*x*+1, −*y*, −*z*+2.
